# Total replacement of soybean meal with alternative plant-based ingredients and a combination of feed additives in broiler diets from 1 day of age during the whole growing period

**DOI:** 10.1016/j.psj.2024.103854

**Published:** 2024-05-16

**Authors:** L. Marchal, A. Bello, G. Archer, E.B. Sobotik, Y. Dersjant-Li

**Affiliations:** ⁎ Danisco Animal Nutrition & Health (IFF), BH Oegstgeest 2342, The Netherlands; † Department of Poultry Science, Texas A&M University, College Station, TX 77843-2472, USA

**Keywords:** β-glucanase, broilers, protease, probiotics, soybean meal

## Abstract

The capacity of combinations of feed enzymes, natural betaine and a probiotic, combined with alternative plant-based ingredients, to totally replace soybean meal (**SBM**) in a broiler diet was evaluated. Day-old Ross 308 males (2,574) were assigned to 9 treatments (13 pens/treatment, 22 birds/pen) in a completely randomized design. All diets were pelleted and fed ad libitum in 4 phases: starter, grower, finisher 1, finisher 2 (0–10, 10–21, 21–35, and 35–42 d of age, respectively). Treatments included: 1) control diet containing SBM (SBM control), supplemented with phytase (PhyG), at 2,000, 1,500, 1000 and 1,000 FTU/kg in each phase and xylanase (**X**) at 750 U/kg, [crude protein (**CP**): 23.5%, 22.0%, 20.2% and 19.3% in each phase]; 2) to 5), alternative (**ALT**), SBM-free diets, containing the same CP level as the control (“CP high”), supplemented with PhyG as in the control, protease (P, 800 U/kg) and in 2) xylanase (750 U/kg) (ALT+PhyG+P+X), 3) xylanase-β-glucanase (XB, 1,200 U/kg and 152 U/kg) (Alt+PhyG+P+XB), 4) XB plus betaine (800 g/ton) (ALT+PhyG+P+XB+Bet), and 5) XB plus a probiotic [150,000 colony forming units (**CFU**)/g] (ALT+PhyG+P+XB+Prob); 6) to 9) as treatments 2) to 5) but with CP reduced by -2.0 to -1.5% points vs. control (‘CP low’). Final (d 42) BW and overall (d 0–42) feed conversion ratio (**FCR**) of birds fed the SBM control exceeded breeder objectives (+3.8% and -1.9%, respectively). Overall FCR was reduced and d 42 BW increased in birds fed “low” vs. “high” CP (*P* < 0.01). Overall FCR and feed intake were not different in ALT+PhyG+XB+P+Bet and ALT+PhyG+XB+P+Prob vs. the control, whereas final BW was reduced (*P* < 0.05) in all ALT treatments but close to breeder objectives (98.3%) in ALT+PhyG+XB+P+Prob. Feed costs of this treatment were similar to the control. Total replacement of SBM with alternative plant-based ingredients in a CP-low diet supplemented with hydrolytic enzymes and probiotics can achieve growth performance outcomes close to commercial breeder objectives.

## INTRODUCTION

Soybean meal (**SBM**) is the major source of protein in broiler diets worldwide. It has a high protein content [crude protein (**CP**) ∼48%], high energy content [metabolizable energy (**ME)** 2,200 Kcal/kg], low fiber content (crude fiber ∼3.6%) and contains an amino acid (**AA**) profile that is well matched to bird requirements (CVB, 2021). However, the cost of SBM is continually increasing (Research and Markets, 2021) and its availability can fluctuate, not only due to cost pressures but also other factors such as those imposed by conflicts or pandemics. Further, in certain producing regions within South America, soy has a high carbon footprint, whilst in non-producing regions (Asia and the EU), there is heavy reliance on imported soy, increasing the carbon footprint further. In light of these factors, there is growing interest in certain regions in using more locally produced alternatives to SBM.

In wholly vegetable diets, SBM-alternatives must be plant-based, deliver a similar digestible AA profile in the total ration to diets based on SBM and exhibit a proven capacity to maintain growth performance when used in commercial diets. Potential alternatives include rapeseed meal (**RSM**; in some regions referred to as canola meal [**CM**; Mushtaq et al., 2007; Xu et al., 2012; Payvastegan et al., 2017]), sunflower meal (**SFM**; Rama Rao et al., 2006; de Morais Oliveira et al., 2016), cottonseed meal (**CSM**; Abdallh et al., 2020), linseed meal (Tamasgen et al., 2021), peas (Bingol et al., 2016) and potato meal (Sultana et al., 2016). Most studies to date have investigated the effect of partial rather than total SBM replacement. Results have been variable. Several studies that have used a variety of CP levels [some above NRC 1994 requirements (Shires et al. 1981), some below requirements with essential AA balanced out via addition of synthetic AA (Ahmad et al., 2007; Mushtaq et al., 2007)] have shown that RSM included at up to 20% of the diet in all phases did not affect growth performance. Meanwhile, in nutritionally adequate diets, Olukosi et al. (2017) observed that increasing the inclusion level of RSM between 0 and 20% of the diet linearly decreased weight gain and increased FCR but maintained overall performance above the breed target, suggesting that RSM inclusion at 20% might be acceptable depending on the desired growth rate in the production system. Payvastegan et al. (2017) similarly noted a linear reduction in BW gain (**BWG**) during 1 to 42 d of age with increasing inclusion of CM from 0 to 30% as a replacement for SBM in nutritionally adequate diets. Meanwhile, Abdallh et al. (2020) reported that CSM could replace 26%, 55% and 100% of SBM during starter, grower and finisher phases, respectvely (6, 12, and 18% CSM in the diet) in broilers up to 35 d of age, whereas Henry et al. (2001) reported impaired performance at 21 d of age with a CSM inclusion level of 20% in the diet (replacing 51% of SBM).

Total SBM replacement is challenging because most plant-based alternatives have a substantially higher fiber content (de Vries and Lannuzel, 2021), including higher associated non-starch polysaccharide (**NSP**) content that can reduce the digestibility of energy, organic matter and nitrogen (Jørgensen et al., 1996) and lead to a lower AA digestibility (Choct et al. 2010). In addition, they typically have a less well-balanced AA profile than SBM. Cottonseed meal and RSM are lower in essential lysine, threonine and tryptophan than SBM (Yun et al., 2017) and SFM is also lower in lysine (González-Pérez, 2015). Supplemental (synthetic) lysine may improve the nutritional value of diets containing RSM, enabling a higher proportion of SBM to be replaced without ill effect (Kocher et al., 2000). Finally, plant-based alternatives also contain anti-nutritional factors (**ANF**) that can impair AA digestibility. These include glucosinolates in RSM that can disrupt thyroid gland functioning and impair growth performance (Tripathi and Mishra, 2007; Payvastegan et al., 2017), gossypol in CSM that can suppress feed intake and growth performance (Henry et al., 2001) and alkaloids and tannins in potato and pea proteins. Heat processing technologies can reduce ANF effects, but the response is not the same for all cereals and pulses and prolonged treatment can reduce rather than increase protein digestibility (del Rio et al., 2022).

Exogenous enzymes and other types of feed additives have proven useful in a variety of conventional dietary settings for increasing nutrient availability and utilization in broilers, and may also be useful for this purpose in SBM-free diets. The capacity of phytase to improve phosphorus (**P**), calcium (**Ca**) and AA digestibility and utilization in broilers has been well established (Selle and Ravindran, 2007; Humer et al., 2015; Dersjant-Li et al., 2022). As a result, it is now added almost ubiquitously to broiler diets, often as a background enzyme on top of which other enzymes or classes of additive are added. Non-starch polysaccharide-degrading enzymes (mainly xylanase and β-glucanase) are increasingly being used in combination with phytase to improve fiber digestion in diets containing RSM, with variable results. Mushtaq et al. (2007) reported no significant effect of a xylanase–β-glucanase combination added to corn and SBM-based diets containing 2 levels of RSM (20% and 30%) on growth performance during 0 to 42 d of age, whereas the study by Abdallh et al. (2020) reported that xylanase–β-glucanase supplementation of diets containing 3 levels of CSM as a replacement for SBM (up to a maximum of 6, 12, and 18% CSM in starter, grower and finisher phases) improved FCR and increased weight gain in grower and finisher phases compared to unsupplemented diets. Meanwhile, protease can improve protein digestibility, particularly in low CP diets (Angel et al., 2011; Mohammadigheisar and Kim, 2018). Certain non-enzyme additives that are effective in supporting gut health and physiology in broilers may also be useful in improving growth performance when more fibrous, complex diets are fed. Specifically, natural betaine, a by-product of the sugar beet industry, exhibits (amongst other beneficial properties) osmolytic activity that acts to increase cell volume thereby contributing to the maintainence of barrier integrity. Betaine has been shown to improve growth performance in heat-stressed-broilers (Shakeri et al., 2018) and to improve BW and BWG in non-heat-stressed birds fed corn, wheat and extracted SBM-based diets when supplemented at 1,000 mg/kg (Awad et al., 2022). Other studies in pigs have indicated that betaine increases the digestibility of fiber in the small intestine as well as bacterial degradation of CP in the large intestine (Ratriyanto et al., 2010) which suggests that it may be beneficial for maintaining performance through enhancing nutrient digestibility in more fibrous diets. Finally, probiotics have a different mode of action to feed enzymes and can support not only gut health but also growth performance in poultry, through increasing digestive enzyme activity, improving feed intake and digestion, stimulating the immune system and beneficially modulating the intestinal microflora (Lee et al., 2010; Jha et al., 2020). *Bacillus-*based probiotics have been shown to improve growth performance, intestinal microbiota, immune responses and fiber hydrolysis (Arif et al., 2021; Gibbs et al., 2021; Vu et al., 2022). It was expected that the addition of a *Bacillus*-based probiotic to a fibrous SBM-free diet in combination with feed enzymes might achieve a further benefit to growth performance due to multiple modes of action.

Against this background, it was hypothesized that the use of exogenous enzymes (protease, xylanase and β-glucanase) in different combinations with betaine and probiotics to enhance nutrient availability and support gut health may enable 100% of SBM to be replaced by plant-based alternatives without loss of growth performance. Phytase was present in all diets as a background enzyme, whereas xylanase was included at a low level in the SBM control diet that contained xylans from the wheat ingredient, and at higher dose level in the SBM-free, alternative, diets that contained additional fibrous ingredients such as rapeseed meal and sunflower seed meal on top of the wheat ingredient. The additive combinations were tested in a SBM-free diet of “high” CP content (i.e., meeting CP specifications for the breed, containing 23.5, 22.0, 20.2, and 19.3% CP during starter, grower and finisher phase, respectively), and in an SBM-free diet of “low” CP content (reduced in CP vs. breed specifications, by 2.0 to 1.5% points vs. “high” but with essential AA balanced out via greater addition of synthetic AA). The “low” CP diet was included because of the growing interest within the poultry industry in such diets due to their potential to reduce N and ammonia emissions, improve litter quality and consequently bird welfare, and reduce feed costs, as recently reviewed by Liu et al. (2021). The overall objective was to determine the effect of the SBM-free diets containing “high” or “low” CP and different combinations of the selected feed additives on growth performance, feed costs and carbon footprint, relative to a comparable diet containing a “high” CP content and SBM as the main protein source.

## MATERIALS AND METHODS

The study was carried out at Texas A&M University (TX, USA). All experimental protocols were reviewed and approved by the Animal Care and Use Committee of Texas A&M University before the research commenced.

### Birds, Housing, and Experimental Design

A total of 2,574 Ross 308 male broilers were obtained on day of hatch from a commercial hatchery where they had been vaccinated against Marek's disease. Birds were assigned to floor-pens (22 birds/pen) on the basis of initial BW so that each pen contained birds of approximately equal BW. Pens were lined with clean (unused) pine wood shavings. There were 13 replicate floor pens per treatment and 9 treatments, in a completely randomized design. Pens were located in an animal house in which temperature was maintained initially at 32ºC and gradually reduced to 23ºC by wk 4. The lighting regime was LD 18:6 h.

### Experimental Diets

Summary details of the 9 dietary treatments are given in Table 1. All diets were formulated using a 4-phase feeding program (starter, 0–10 d of age; grower, 10–21 d of age; finisher 1, 21–35 d of age, and; finisher 2, 35–42 d of age).

**Table 1:** Treatment details.

Treatment	CP level^1^	Phytase^2^^,^^3^ in starter, grower, finisher, FTU/kg	Xylanase^2^^,^^4^ in all phases, U/kg	β-glucanase^2^^,^^4^ in all phases, U/kg	Protease^2^^,^^5^ in all phases, U/kg	Natural Betaine^2^^,^^6^ in all phases, g/ton	Probiotic^7^ in all phases, CFU/g
SBM +PhyG+X (control)	“high”	2,000 / 1,500 / 1,000	750	-	-	-	-
ALT+PhyG+X+P	“high”	2,000 / 1,500 / 1,000	750	-	800	-	-
ALT+PhyG+XB+P	“high”	2,000 / 1,500 / 1,000	1,200	152	800	-	-
ALT+PhyG+XB+P+Bet	“high”	2,000 / 1,500 / 1,000	1,200	152	800	800	-
ALT+PhyG+XB+P+Prob	“high”	2,000 / 1,500 / 1,000	1,200	152	800	-	150,000
ALT+PhyG+X+P	“low”	2,000 / 1,500 / 1,000	750	-	800	-	-
ALT+PhyG+XB+P	“low”	2,000 / 1,500 / 1,000	1,200	152	800	-	-
ALT+PhyG+XB+P+Bet	“low”	2,000 / 1,500 / 1,000	1,200	152	800	800	-
ALT+PhyG+XB+P+Prob	“low”	2,000 / 1,500 / 1,000	1,200	152	800	-	150,000

1 ′high’, 23.5%, 22.0%, 20.2% and 19.3% in starter, grower, finisher 1 and finisher 2 phase, respectively; ‘low’, reduced by -2.0 to -1.5% points vs. ‘high’, across phases.

2 Stated activity levels of enzymes are formulated levels.

3 A novel consensus bacterial 6-phytase variant (PhyG) produced in *T. reesei,* Axtra PHY GOLD (Danisco Animal Nutrition & Health, IFF).

4 A commercial endo-1.4-ß-xylanase (EC 3.2.1.8), Danisco Xylanase (Danisco Animal Nutrition & Health, IFF), originating from *Trichoderma reesei*, or a combination of this xylanase with a commercial endo-1,3(4) – beta glucanase (EC 3.2.1.6) produced in *T. reesei*, as a co-granule (Axtra XB; Danisco Animal Nutrition & Health, IFF), according to treatment.

5 A commercial serine protease (EC 3.4.21.62) originating from *Bacillus subtilis*, Axtra PRO (Danisco Animal Nutrition & Health, IFF).

6 A commercial anhydrous natural betaine, Betafin (Danisco Animal Nutrition & Health, IFF).

7 A commercial probiotic based on spores of a combination of 3 strains of *B. velezensis,* Enviva PRO (Danisco Animal Nutrition & Health, IFF). ALT, alternative (SBM-free); Bet, betaine; CP, crude protein; P, protease; Prob, probiotic; PhyG, novel consensus bacterial 6-phytase variant; SBM, soybean meal; XB, xylanase–β-glucanase; X, xylanase.

The control diet contained SBM, wheat and corn with a small amount of added RSM and SFM and was supplemented with phytase and a low level of xylanase. The phytase was a novel consensus bacterial 6-phytase variant (**PhyG;** Danisco Animal Nutrition & Health, IFF) produced in *Trichoderma reesei* and added at 2,000, 1,500, 1,000 and 1,000 phytase units (**FTU**) per kilogram of feed during starter, grower, finisher 1 and finisher 2 phases, respectively. [This tiered-byphase dosing regimen was employed primarily because previous research on this phytase in broilers (Marchal et al., 2021) has shown that a tiered dosing regimen can result in an additional degree of improvement in weight gain and feed conversion efficiency compared to a constant, lower, dose of phytase, due to the higher nutrient requirements of younger relative to older birds. In addition, it was considered that having a higher dose level of the phytase in starter phase when AA requirements are high, might improve AA digestibility in the alternative plant-ingredients, given that it has been shown that PhyG can improve AA digestibility in a dose-dependent manner (Dersjant-Li et al., 2022)]. The xylanase (**X**) was an endo-1.4-ß-xylanase (EC 3.2.1.8) originating from *Trichoderma reesei,* added at 750 xylanase units (**U**)/kg during all phases. The digestible P and calcium content of the control diet was reduced according to the expected contribution of the phytase based on the dose level applied per phase. The CP content of the control diet was formulated at 23.5, 22.0, 20.2, and 19.3% in starter, grower, finisher 1 and finisher 2, respectively. These levels approximately met the recommended CP levels set by the breeder when the trial took place (2021).

Two alternative (**ALT**), SBM-free diets were formulated by replacing SBM with additional RSM and SFM and commercially available sources of pea protein, pea flour, CSM, potato protein and corn gluten meal. One ALT diet was formulated to contain the same CP level as the SBM control (“high” CP), whereas the other was reduced in CP by 2.0 to 1.5% points vs. the control (“low” CP). Levels of the first 8 limiting AA were maintained equal in “high” and “low” CP diets by increasing the content of synthetic AA in the CP “low” diet. Both ALT diets were supplemented with PhyG as in the SBM control as well as either 1) an exogenous serine protease added at 800 U/kg and xylanase at 750 U/kg (ALT+PhyG+P+X), 2) protease at 800 U/kg and a commercial xylanase–β-glucanase, added to provide 1,200 U/kg xylanase and 152 U/kg β-glucanase (ALT+PhyG+P+XB), 3) the same level of protease, xylanase–β-glucanase and a natural betaine (**Bet**) added at 800 g/ton (ALT+PhyG+P+XB+Bet) or; d) the same level of protease, xylanase–β-glucanase and a probiotic based on spores of a combination of 3 strains of *Bacillus velezensis* (formerly *B. amyloliquefaciens*), added at 150,000 colony forming units (**CFU**)/g feed (ALT+PhyG+P+XB+Prob). All feed additives were manufactured by and obtained from Danisco Animal Nutrition & Health (IFF), the Netherlands.

The full ingredient and nutrient compositions of the SBM control and ALT diets is presented in Table 2. The analyzed nutrient content of the plant-based alternatives to SBM is presented in Table. 3.

**Table 2:** Ingredient and calculated nutrient composition of the basal diets^1^ (as fed basis), by phase.

	Starter (0–10 d of age)			Grower (10–21 d of age)			Finisher 1 (21–35 d of age)			Finisher 2 (35–42 d of age)		
	SBM (control)	ALT “high” CP	ALT “low” CP^2^	SBM (control)	ALT “high” CP	ALT “low” CP^2^	SBM (control)	ALT “high” CP	ALT “low” CP^2^	SBM (control)	ALT “high” CP	ALT “low” CP^2^
Ingredients, %												
Wheat (13.9% CP)	40.4	36.3	41.0	42.5	38.4	43.7	47.4	44.3	52.5	48.9	46.5	52.0
Corn (6.92% CP)	20.0	20.0	20.0	20.0	20.0	20.0	20.0	20.0	20.0	20.0	20.0	20.0
Soybean meal (44.8% CP)	34.1	-	-	28.3	-	-	22.1	-	-	18.1	-	-
Rapeseed meal (38.8% CP)	1.2	7.1	7.1	2.4	10.0	10.0	3.1	10.0	10.1	4.3	10.0	10.2
Sunflower meal (27.8% CP)	1.1	2.1	2.4	2.4	2.0	2.0	2.8	1.2	1.0	3.8	1.5	2.0
Pea protein (50.0% CP)	-	7.8	7.7	-	3.3	2.3	-	4.1	1.1	-	3.5	1.7
Pea flour (22.4% CP)	-	7.3	6.9	-	6.9	6.5	-	5.1	2.3	-	4.3	2.2
Decorticated cottonseed meal (43.4% CP)	-	6.1	6.0	-	5.9	5.3	-	5.0	4.5	-	5.0	4.3
Potato protein (75.2% CP)	-	3.7	1.4	-	3.2	1.5	-	2.0	1.1	-	1.6	0.5
Corn gluten meal (64.4% CP)	-	3.6	1.6	-	3.8	2.0	-	2.1	1.2	-	1.4	0.8
Rapeseed oil	0.8	1.9	1.1	2.1	3.1	2.3	2.5	3.1	2.2	3.1	3.6	2.9
Limestone	0.6	1.3	1.2	0.6	1.1	1.1	0.7	1.0	1.0	0.6	0.9	0.9
Monocalcium phosphate	0.4	0.5	0.5	0.2	0.2	0.2	0.1	0.1	0.1	-	-	-
DL-methionine	0.3	0.3	0.4	0.3	0.2	0.3	0.2	0.2	0.3	0.2	0.2	0.2
L-lysine HCL	0.2	0.3	0.5	0.2	0.4	0.6	0.2	0.3	0.6	0.3	0.3	0.5
L-threonine	0.1	0.2	0.3	0.1	0.1	0.2	0.1	0.1	0.2	0.1	0.1	0.2
L-tryptophan	-	-	-	-	-	-	-	-	-	-	-	-
L-isoleucine	-	-	0.2	-	-	0.2	-	-	0.2	0.0	0.1	0.2
L-valine	-	-	0.1	-	-	0.1	-	-	0.1	-	-	0.1
L-arginine	-	-	0.1	-	-	-	-	-	0.2	-	-	0.1
Potassium carbonate	-	0.7	0.7	-	0.6	0.7	-	0.4	0.6	-	0.3	0.4
Vitamin-mineral premix^3^	0.3	0.3	0.3	0.3	0.3	0.3	0.3	0.3	0.3	0.3	0.3	0.3
Sodium bicarbonate	0.3	0.3	0.4	0.3	0.3	0.4	0.2	0.2	0.3	0.3	0.2	0.3
Sodium chloride	0.2	0.1	0.1	0.2	0.1	0.1	0.2	0.1	0.1	0.2	0.1	0.1
Calculated nutrients, % unless otherwise stated												
Metabolizable energy, kcal/kg	2,950	2,950	2,950	3,050	3,050	3,050	3,100	3,100	3,100	3,150	3,150	3,150
Crude fat	2.51	4.49	3.74	4	6	5	4.55	5.78	4.79	5.32	6.26	5.59
Crude fiber	3.38	6.41	6.01	3.89	6.74	6.29	4.08	6.09	5.67	4.49	6.00	5.81
Ash	4.27	4.58	4.67	4.06	4.33	4.39	3.77	3.98	4.03	3.59	3.75	3.82
Starch	37.64	38.88	41.12	38.86	39.96	42.60	41.68	42.37	45.87	42.57	43.20	45.48
Calcium^5^	0.69	0.69	0.69	0.62	0.62	0.62	0.56	0.56	0.56	0.50	0.50	0.50
dig. Phosphorus^4^	0.24	0.24	0.24	0.19	0.19	0.19	0.17	0.17	0.17	0.15	0.15	0.14
Phytate phosphorus	0.28	0.27	0.27	0.28	0.29	0.29	0.28	0.28	0.28	0.28	0.28	0.28
Total phosphorus	0.52	0.55	0.55	0.46	0.49	0.48	0.42	0.45	0.44	0.41	0.43	0.42
Sodium	0.15	0.15	0.15	0.15	0.15	0.15	0.14	0.14	0.14	0.14	0.14	0.14
Chloride	0.20	0.20	0.20	0.20	0.20	0.20	0.20	0.20	0.20	0.20	0.20	0.20
Potassium	0.99	0.99	0.99	0.90	0.90	0.90	0.80	0.80	0.80	0.74	0.74	0.74
DEB	261	261	261	239	239	239	209	209	209	194	194	194
Crude protein	23.53	23.53	21.53	22.00	22.00	20.00	20.24	20.22	18.22	19.29	19.29	17.79
dig. AA (sum of 18)	21.80	23.35	20.51	20.18	20.64	17.62	18.25	18.98	15.45	17.20	17.84	15.26
SID Lys	1.22	1.22	1.22	1.11	1.11	1.11	0.99	0.99	0.99	0.93	0.93	0.93
SID Met & cys	0.90	0.90	0.90	0.84	0.84	0.84	0.77	0.77	0.77	0.73	0.73	0.73
SID Met	0.59	0.61	0.63	0.54	0.55	0.57	0.50	0.50	0.52	0.46	0.46	0.47
SID Thr	0.81	0.81	0.81	0.74	0.74	0.74	0.65	0.65	0.65	0.62	0.62	0.62
SID Trp	0.25	0.20	0.20	0.22	0.18	0.18	0.20	0.17	0.16	0.19	0.16	0.15
SID Arg	1.37	1.34	1.31	1.25	1.20	1.20	1.10	1.10	1.06	1.02	1.04	1.01
SID Ile	0.84	0.81	0.81	0.76	0.75	0.75	0.67	0.67	0.67	0.64	0.64	0.64
SID Leu	1.53	1.64	1.35	1.41	1.54	1.26	1.27	1.33	1.08	1.19	1.22	1.03
SID Val	0.91	0.91	0.91	0.84	0.84	0.84	0.75	0.75	0.75	0.71	0.71	0.71
SID Ala	0.85	0.93	0.76	0.79	0.88	0.71	0.70	0.75	0.60	0.66	0.69	0.58
SID Asp	1.87	1.59	1.37	1.67	1.39	1.17	1.44	1.24	0.95	1.31	1.16	0.94
SID Cys	0.30	0.29	0.26	0.29	0.29	0.26	0.27	0.27	0.26	0.27	0.26	0.25
SID Glu	4.09	3.89	3.59	3.85	3.69	3.37	3.59	3.48	3.15	3.44	3.36	3.13
SID Gly	0.79	0.75	0.66	0.73	0.70	0.62	0.66	0.64	0.55	0.63	0.61	0.54
SID His	0.51	0.47	0.42	0.47	0.44	0.39	0.42	0.40	0.34	0.40	0.38	0.34
SID Phe	0.99	1.01	0.85	0.91	0.92	0.77	0.81	0.82	0.67	0.76	0.76	0.64
SID Pro	1.23	1.24	1.09	1.17	1.22	1.08	1.11	1.12	1.03	1.07	1.07	1.00
SID Ser	0.97	0.92	0.79	0.89	0.84	0.73	0.80	0.77	0.64	0.75	0.72	0.62
SID Tyr	0.69	0.71	0.58	0.62	0.64	0.52	0.55	0.56	0.44	0.51	0.52	0.42
Soluble arabinoxylans	0.93	0.90	0.95	0.94	0.96	1.02	0.98	1.00	1.07	0.99	1.02	1.06
Total arabinoxylans	4.59	4.46	4.69	4.70	4.73	4.95	4.84	4.82	5.10	4.93	4.91	5.14
Soluble beta-glucans	0.47	0.39	0.42	0.47	0.42	0.45	0.48	0.45	0.49	0.49	0.46	0.49
Total beta-glucans	3.09	3.14	3.27	3.15	3.36	3.45	3.15	3.29	3.32	3.22	3.33	3.39
Total beta-mannans	0.12	0.16	0.17	0.14	0.18	0.19	0.15	0.20	0.22	0.17	0.20	0.22
Insoluble NSPs	8.47	6.16	6.53	8.45	6.73	7.09	8.30	6.82	7.12	8.33	6.95	7.30
Soluble NSPs	2.39	1.81	1.92	2.36	1.97	2.09	2.32	2.02	2.11	2.31	2.05	2.13
Total NSPs	13.89	13.33	13.50	13.87	13.90	13.90	13.52	13.54	13.34	13.59	13.59	13.60
Total CFP, g CO_2_/kg^5^	1,879	727	677	1,646	658	644	1,246	659	652	1,222	614	636
CFP, fossil fuels^5^	484	547	490	472	496	513	467	501	479	455	481	469

1 Exclusive of feed additives that were added post-analysis, according to treatment.

2 CP -2.0 to -1.5% points vs. the SBM diet, dependent on the phase.

3 Vitamin premix added at this rate of 8,818 IU vitamin A, 3,086 IU vitamin D3, 37 IU vitamin E, 0.0132 mg B12, 4.676 mg riboflavin, 36.74 mg niacin, 16.17 mg pantothenic acid, 382.14 mg choline, 1.18 mg menadione, 1.4 mg folic acid, 5.74 mg pyridoxine, 2.35 mg thiamine, 0.44 mg biotin per kg diet and trace mineral premix added at this rate of 149.6 mg manganese, 125.1 mg zinc, 16.5 mg iron, 1.7 mg copper, 1.05 mg iodine, 0.25 mg selenium, a minimum of 6.27 mg calcium, and a maximum of 8.69 mg calcium per kg of diet. The carrier is calcium carbonate and the premix contain less than 1% mineral oil.

4 Exclusive of the expected contribution of the added phytase.

5 Calculated using Wageningen FeedPrint NL software (FeedPrint NL, 2020). ‘Total CFP. includes the estimated CFP from fossil fuels and from land use and land use change, whereas “CFP, fossil fuels” includes the estimated CFP only from the associated use of fossil fuels in the production of the diet.’ ALT, alternative (soybean-free); CFP, carbon footprint; DEB, dietary electrolyte balance; SBM, soybean meal; SID, standardized ileal digestible.

**Table 3:** Analyzed composition of plant-protein ingredients used in the study, % as is.

Item	Soybean meal	Rapeseed meal	Sunflower meal	Pea protein	Pea flour	Potato protein	Cottonseed meal^1^	Corn gluten meal
Moisture	12.28	7.52	5.97	10.94	11.49	9.82	11.69	9.38
DM	87.72	92.48	94.03	89.06	88.51	90.18	88.31	90.62
CP	44.80	34.10	27.80	50.00	22.40	75.20	43.40	64.40
Crude fat	1.18	9.73	10.30	3.26	1.00	0.97	3.02	2.97
Acid detergentfiber	3.46	20.00	29.00	3.60	6.80	16.40	18.40	9.80
Ash	6.77	6.17	5.57	5.11	3.00	0.23	5.96	1.45
Total P	0.65	1.03	0.92	0.82	0.41	0.12	1.08	0.50
K	2.15	1.18	1.26	1.84	1.04	0.02	1.54	0.17
Ca	0.98	0.83	0.38	0.16	0.11	0.02	0.30	0.01
Na	0.01	-	-	-	-	-	0.26	0.06

1 Decorticated.

Additives were premixed with ground corn before adding to the main batch of the basal diet which was then further mixed to ensure a homogenous distribution within the diet. Following mixing, diets were pelleted at a temperature of 80 to 85ºC. All diets were provided to birds ad libitum. Drinking water was freely available for the duration of the study.

### Measurements, Sampling, and Chemical Analysis

Birds were monitored daily for mortality and dead birds were removed and weighed. Pen BW and feed intake (**FI**) were measured at 0, 10, 21, 35, and 42 d of age and used to calculate BWG and feed conversion ratio (**FCR**), corrected for mortality.

Representative sub-samples of all final diets were analyzed for proximate components and minerals. Nutrients were analyzed according to the following methods: crude protein, NEN-EN-ISO 16634 (ISO, 2008); crude fat, NEN-ISO 6492 (ISO, 1999); crude fiber, NEN-ISO 6865 (ISO, 2000). Phosphorus, Ca, K, and Na were analyzed by microwave digestion and Inductively Coupled Plasma-Optical Emission Spectrometry (**ICP-OES**) in accordance with method AOAC 2011.14 (AOAC, 2011). Ash was determined by dry ashing in a muffle furnace according to AOAC method 945.02 (AOAC, 2005) and gross energy was determined by bomb calorimetry (AOAC, 1990).

### Calculations

Total feed costs per kilogram of bodyweight gain (averaged over the overall period, 0–42 d of age) were calculated using ingredient and feed additive prices in operation in June 2021.

An estimate of the total carbon footprint (**CFP**) of the treatment diets was made using Wageningen FeedPrint NL software (FeedPrint NL, 2020) including that from fossil fuels, land use and land use change, as well as for fossil fuel CFP only, and are presented in Table 2. The calculations were combined with measurements of feed intake and BWG in each phase to estimate the total carbon footprint of the diets per kilogram of BWG.

### Statistical Analysis

Data are reported per pen as the experimental unit. Data were analyzed by one-way ANOVA to examine the effects of treatment on response measures and by 2-way ANOVA (2×4 factorial analysis with the SBM control excluded) to test the effect of 1) CP level and 2) feed additive combination, on response measures. Means separation was achieved using Tukey's HSD test, with a 95% confidence interval applied. All analyses were conducted using the Fit Model Platform of JMP 15.0 (JMP, 2020). Differences were considered significant at *P* < 0.05.

## RESULTS

The analyzed nutrient content of the experimental diets is presented in Table 4.

**Table 4:** Analyzed nutrient (formulated values in brackets) concentrations (as-fed basis) of the basal SBM, and ALT diets, % unless otherwise stated.

Phase	Basal diet	Moisture	DM	CP	Crude fat	ADF (crude fiber)	Ash	Phosphorus (total)	K	Ca	Na
Starter (0 to 10 d of age)	SBM (control)	13.09	86.91	24.31 (23.53)	3.67 (2.51)	4.00 (3.38)	4.53 (4.27)	0.54 (0.51)	1.04(0.99)	0.58 (0.69)	0.16 (0.15)
	ALT “high” CP	12.77	87.23	21.81 (23.52)	4.60 (4.49)	5.30 (6.41)	4.72 (4.58)	0.57 (0.55)	0.91(0.99)	0.73 (0.69)	0.16 (0.15)
	ALT “low” CP^1^	13.45	86.55	20.06 (25.53)	3.75 (3.74)	5.10 (6.01)	4.31 (4.67)	0.55 (0.55)	0.94(0.99)	0.64 (0.69)	0.14 (0.15)
Grower (10 to 21 d of age)	SBM (control)	15.09	84.91	21.10 (22.00)	3.37 (4.00)	4.80 (3.89)	3.84 (4.06)	0.49 (0.46)	0.95(0.90)	0.51 (0.62)	0.15 (0.15)
	ALT “high” CP	14.71	85.29	20.80 (22.00)	4.51 (6.00)	5.60 (6.74)	4.10 (4.33)	0.50 (0.49)	0.87(0.90)	0.66 (0.62)	0.16 (0.15)
	ALT “low” CP^1^	14.29	85.71	18.00 (20.00)	3.96 (5.00)	5.20 (6.29)	3.70 (4.39)	0.48 (0.48)	0.89(0.90)	0.60 (0.62)	0.15 (0.15)
Finisher 1 (21 to 35 d of age)	SBM (control)	12.48	87.52	20.44 (20.24)	4.37 (4.55)	4.30 (4.08)	3.86 (3.77)	0.46 (0.42)	0.89(0.80)	0.55 (0.56)	0.17 (0.14)
	ALT “high” CP	12.40	87.60	20.00 (20.22)	5.44 (5.78)	6.10 (6.09)	4.07 (3.98)	0.49 (0.45)	0.82 (0.80)	0.71 (0.56)	0.15 (0.14)
	ALT “low” CP^1^	13.10	86.90	17.20 (18.22)	3.98 (4.79)	4.90 (5.67)	3.70 (4.03)	0.45 (0.44)	0.79 (0.80)	0.71 (0.56)	0.16 (0.14)
Finisher 2 (35 to 42 d of age)	SBM (control)	12.54	87.46	19.56(19.29)	4.59 (5.32)	4.40 (4.49)	3.58 (3.59)	0.44 (0.41)	0.81 (0.74)	0.46 (0.50)	0.15 (0.14)
	ALT “high” CP	12.25	87.75	18.88 (19.25)	5.05 (6.26)	5.60 (6.00)	3.61 (3.75)	0.47 (0.43)	0.75 (0.74)	0.53 (0.50)	0.16 (0.14)
	ALT “low” CP^1^	13.32	86.68	17.50 (19.79)	4.63 (5.59)	5.60 (5.81)	3.83 (3.82)	0.39 (0.42)	0.68 (0.74)	0.57 (0.50)	0.15 (0.14)

1 formulated with -2.0 to -1.5% points CP vs. SBM (control) diet. ADF, acid-detergent fiber; SBM, soybean meal; ALT, alternative (SBM-free); CP, crude protein The analyzed phytase activity across treatments are: 2716; 2698; 1440 and 1352 FTU/kg in starter, grower, finisher 1 and finisher 2 respectively. The analyzed xylanase activity across phases are: 1370; 806; 1046; 1785; 1518; 1820; 1730; 1738; 1685 U/kg respectively for diets 1, 2, 3, 4, 5, 6, 7, 8, 9 (analysis was performed by Eurofins, USA).

### Growth Performance

Effects of treatment on growth performance, analyzed by one-way ANOVA, are presented by phase in Table 5 and cumulatively in Table 6.

**Table 5:** Effect of treatment^1^ growth performance, by phase; results of one-way ANOVA.

	Treatments^1^										
	‘high’ crude protein					‘low’ crude protein^2^					
Item	SBM PhyG X (control)	ALT PhyG X P	ALT PhyG XB P	ALT PhyG XB P Bet	ALT PhyG XB P Prob	ALT PhyG X P	ALT PhyG XB P	ALT PhyG XB P Bet	ALT PhyG XB P Prob	SEM	*P*-value
**Starter** (0 to 10 d of age)											
d 10 BW, g/bird	325^a^	294^c^	301^bc^	296^bc^	297^bc^	300^bc^	298^bc^	297^bc^	308^b^	2.73	<0.001
BWG, g/bird	282^a^	251^c^	258^bc^	253^bc^	254^bc^	257^bc^	254^bc^	254^bc^	265^b^	2.67	<0.001
FI, g/bird	303^a^	281^c^	284^c^	283^c^	289^bc^	298^ab^	293abc	291abc	297^ab^	3.03	<0.001
FCR, g:g	1.077^d^	1.126abc	1.101^cd^	1.119^bc^	1.144^ab^	1.164^a^	1.155^ab^	1.147^ab^	1.129abc	0.009	<0.001
**Grower** (10 to 21 d of age)											
d 21 BW	1,111^a^	959^c^	964^c^	976^c^	992^bc^	982^bc^	1,012^bc^	994^bc^	1,035^b^	12.34	<0.001
BWG, g/bird	787^a^	665^cd^	663^d^	679bcd	694bcd	682bcd	715^bc^	697bcd	728^b^	11.43	<0.001
FI, g/bird	1,073^a^	995^bc^	984^c^	976^c^	998^bc^	1,016^bc^	1,034^ab^	984^c^	1,038^ab^	9.66	<0.001
FCR, g:g	1.369^b^	1.504^a^	1.485^a^	1.442^ab^	1.445^ab^	1.494^a^	1.461^ab^	1.423^ab^	1.432^ab^	0.02	<0.001
**Finisher 1** (21 to 35 d of age)											
d 35 BW	2,492^a^	2,123^d^	2,155^cd^	2,153^cd^	2,199^cd^	2,171^cd^	2,236^bc^	2,214^cd^	2,324^b^	23.41	<0.001
BWG, g/bird	1,381^a^	1,165^c^	1,191^c^	1,178^c^	1,208^bc^	1,189^c^	1,224^bc^	1,220^bc^	1,288^ab^	20.58	<0.001
FI, g/bird	2,186^a^	2,107^ab^	2,096^ab^	2,050^b^	2,094^ab^	2,054^b^	2,115^ab^	2,047^b^	2,114^ab^	22.18	<0.001
FCR, g:g	1.599^b^	1.820^a^	1.776^a^	1.765^a^	1.760^a^	1.773^a^	1.750^ab^	1.693^ab^	1.667^ab^	0.035	<0.001
**Finisher 2** (35 to 42 d of age)											
d 42 BW	3,256^a^	2,852^d^	2,853^d^	2,914^cd^	2,964^cd^	2,930^cd^	2,968^c^	2,972^bc^	3,082^b^	5.36	<0.001
BWG, g/bird	765	729	699	761	765	759	732	758	758	22.4	0.43
FI, g/bird	1,423^a^	1,376^ab^	1,310^b^	1,366^ab^	1,385^ab^	1,342^ab^	1,344^ab^	1,363^ab^	1,369^ab^	19.28	0.012
FCR, g:g	1.921	1.939	1.913	1.865	1.852	1.893	1.912	1.868	1.873	0.049	0.932

1 Full treatment details are listed in Table 1.

2 -2.0 to -1.5% points CP vs SBM (control) diet.

a,b,c,d Means within a row bearing different superscript letters are significantly different at *P* < 0.05. Bet, betaine; BWG, BW gain; FI, feed intake; FCR, feed conversion ratio; PhyG, novel consensus bacterial 6-phytase variant; P, protease; Prob, probiotic; X, xylanase; XB, xylanase–β-glucanase

**Table 6:** Effect of treatment^1^ on growth performance, cumulatively, and comparison of feed costs and carbon footprint of the diets; results of one-way ANOVA.

	Treatments										
	‘high’ crude protein					‘low’ crude protein^2^					
Item	SBM PhyG X (control)	ALT PhyG X P	ALT PhyG XB P	ALT PhyG XB P Bet	ALT PhyG XB P Prob	ALT PhyG X P	ALT PhyG XB P	ALT PhyG XB P Bet	ALT PhyG XB P Prob	SEM	*P*-value
**0 to 21 d of age**											
BWG, g/bird	1,068^a^	915.6^c^	920.7^c^	932.6^c^	948.5^bc^	939.0^bc^	969.1^bc^	951.2^bc^	992.4^b^	12.32	<0.001
FI, g/bird	1,370^a^	1,276^cd^	1,267^cd^	1,259^d^	1,287bcd	1,314^bc^	1,326^ab^	1,275^cd^	1,335^ab^	11.09	<0.001
FCR, g:g	1.291^b^	1.399^a^	1.377^a^	1.355^ab^	1.363^a^	1.403^a^	1.378^a^	1.348^ab^	1.351^ab^	0.014	<0.001
**0 to 35 d of age**											
BWG, g/bird	2,449^a^	2,080^d^	2,111^cd^	2,111^cd^	2,156^cd^	2,127^cd^	2,193^bc^	2,171^cd^	2,281^b^	23.28	<0.001
FI, g/bird	3,562^a^	3,383bcd	3,363bcd	3,309^d^	3,381bcd	3,369bcd	3,441abc	3,322^cd^	3,449^ab^	27.67	<0.001
FCR, g:g	1.462^c^	1.634^a^	1.601^ab^	1.580^ab^	1.583^ab^	1.602^ab^	1.579^ab^	1.541^bc^	1.528^bc^	0.018	<0.001
**0 to 42 d of age**											
BWG, g/bird	3,213^a^	2,809^d^	2,810^d^	2,871^cd^	2,921^bc^	2,887^cd^	2,925^c^	2,929^bc^	3,039^b^	25.36	<0.001
FI, g/bird	4,985^a^	4,759^b^	4,673^b^	4,676^b^	4,766^b^	4,710^b^	4,786^b^	4,685^b^	4,818^ab^	39.94	<0.001
FCR, g:g	1.566^d^	1.710^a^	1.676^ab^	1.647^bc^	1.647^bc^	1.661abc	1.658abc	1.622bcd	1.610^cd^	0.014	<0.001
Feed cost^3^ during 0 to 42 d of age, euros/kg BWG	0.584^e^	0.658^a^	0.646^ab^	0.634abc	0.634abc	0.626bcd	0.625bcd	0.611^cd^	0.607^de^	0.006	<0.001
CFP^4^, g CO_2_/kg BWG	2,136^a^	1,111^b^	1,075^bc^	1,070^bc^	1,050^c^	1,090^b^	1,073^bc^	1,070^bc^	1,043^c^	10.67	<0.001

1 Full treatment details are listed in Table 1.

2 −2.0 to −1.5% points CP vs SBM (control) diet.

3 Calculated based on market prices for ingredients in June 2021, inclusive of the cost of the feed additives added according to treatment.

4 CFP, total carbon footprint of the diet per kg BWG, including that from fossil fuels, land use and land use change, was calculated using Wageningen FeedPrint NL software (FeedPrint NL, 2020), taking account of actual feed intake and BWG.

a,b,c,d Means within a row bearing different superscript letters are significantly different at *P* < 0.05. Bet, betaine; BWG, BW gain; FI, feed intake; FCR, feed conversion ratio; PhyG, novel consensus bacterial 6-phytase variant; P, protease; Prob, probiotic; X, xylanase; XB, xylanase–β-glucanase.

There were significant effects of treatment on all outcome measures (BW, BWG, FI, and FCR) during starter, grower and finisher phases (*P* < 0.001 in all cases; Table 5). During starter phase, compared with the SBM control, all ALT diets resulted in reduced d 10 BW, reduced BWG and increased FCR (*P* < 0.05), except in ALT+PhyG+P+XB “low” CP where FCR was not different from that of the SBM control. Feed intake was reduced in ALT “high” CP treatments (*P* < 0.05) but in ALT “low” CP treatments was not different from the SBM control. During grower phase, d 21 BW and 10 to 21 d of age BWG responses were similar to those observed during starter phase (lower in ALT treatments), whilst FCR was increased (*P* < 0.05) in ALT+PhyG+P+X “high” and “low” CP treatments, and in ALT+PhyG+P+XB ‘normal CP, but not different from the SBM control in ALT+PhyG+P+XB “low” CP, ALT+PhyG+P+XB+Bet “high” and “low” CP treatments and ALT+PhyG+P+XB+Prob “high” and “low” CP treatments. During this period, FI intake was reduced (*P* < 0.05) in the majority of ALT treatments but maintained equivalent to the SBM control in ALT+PhyG+P+XB “low” CP and ALT+PhyG+P+XB+Prob “low” CP. During finisher 1 phase, all treatments except ALT+PhyG+P+XB+Prob exhibited reduced (*P* < 0.05) BWG and d 35 BW compared to the SBM control, whereas FCR was maintained similar to the SBM control in ALT+PhyG+XB+P, ALT+PhyG+XB+P+Bet and ALT+PhyG+XB+P+Prob “low” CP and increased in other ALT treatments (*P* < 0.05). During this period, FI was reduced (*P* < 0.05) in ALT+PhyG+P+XB+Bet “high” CP, ALT+PhyG+P+X “low” CP and ALT+PhyG+P+XB+Bet ‘low’ CP but similar to the SBM control in other ALT treatments. During 35 to 42 d of age, BWG and FCR did not differ among treatments. Final (d 42) BW was reduced (*P* < 0.05) in all ALT treatments compared to the SBM control but the reduction was less in ALT+PhyG+P+XB+Prob “low” CP than in any other ALT treatment (*P* < 0.05). This treatment achieved a final BW of 3,082 g/bird in comparison to 3,256 g/bird in the SBM control (*P* < 0.05). Feed intake during this phase did not differ among treatments except in ALT+PhyG+P+XB “low” CP in which it was reduced compared to the SBM control (*P* < 0.05). Cumulatively (Table 6), during 1 to 21 and 1 to 35 d of age, BWG responses followed similar patterns to those observed during 0 to 10 d of age but the FCR response was different. During both periods, treatment ALT+PhyG+P+XB+Prob “low” CP maintained FI and FCR equivalent to the SBM control whereas in most other ALT treatments FI was reduced and FCR was increased (*P* < 0.05). For the overall period (0–42 d of age), BWG was reduced (*P* < 0.05) in all ALT treatments compared with the SBM control but the reduction was smaller (*P* < 0.05) in ALT+PhyG+P+XB+Prob “low” CP than all other ALT treatments except ALT+PhyG+P+XB+Prob “high” CP; overall BWG in ALT+PhyG+P+XB+Prob “low” CP was 174 g/bird below that of the SBM control (3,039 vs. 3,213 g, respectively). In addition, overall FCR was similar to the SBM control in ALT+PhyG+P+XB+Bet and ALT+PhyG+P+XB+Prob “low” CP treatments and FI was similar to control in the ALT+PhyG+P+XB+Prob “low” CP treatment. Mortality was not affected by treatment during any phase or overall (data not shown).

Main effects of dietary CP level (excluding the SBM control from the analysis) and feed additive combination on BW, FI and FCR, analyzed by 2-way ANOVA, are shown in Table 7. There were no interactions between factors except on FCR during d 0 to 10 (*P* < 0.01) where the effect of the feed additive combinations was different in “high” and “low” CP diets. The individual treatment means for “low” CP diets during d 0 to 10 (Table 5) suggest that FCR was reduced in a stepwise manner by each additional additive included in the diet (from X to XB to XB plus Bet or XB plus Prob) but remained similar or increased with each additional additive added to the diet in “high” CP diets. Across additive combinations, the main effect of dietary CP level in “low” relative to “high” CP diets was to increase FI during 0 to 10 and 0 to 21 d of age (+3.9% and +3.0%, respectively; *P* < 0.001) and to increase BW from d 21 onwards [by 3.5% (*P* < 0.01) at d 21, 3.6% (*P* < 0.001) at d 35 and 3.2% (*P* < 0.001) at d 42; Table 7] and to decrease FCR during d 0 to 35 and d 0 to 42 [by 4 FCR points (*P* < 0.01) during d 0 to 35 and 3 FCR points (*P* < 0.01) during d 0 to 42; Table 7]. Across CP levels, significant main effects of feed additive combination on BW, FI, and FCR were also observed. At each of d 21, 35 and 42, BW was greater (*P* < 0.05) in ALT treatments containing XB plus the probiotic than those containing only X (plus the phytase and protease that were in all ALT treatments). At d 35, BW in ALT treatments containing XB plus the probiotic was also greater than in treatments containing XB plus betaine, and at d 42 BW it was also greater than in treatments containing only XB (*P* < 0.05). During 10 to 21 d of age, FI was higher in ALT treatments containing X or XB plus the probiotic than in the treatmentcontaining XB plus betaine. In relation to FCR, this was lower in ALT treatments containing XB plus probiotics or XB plus betaine than in treatments containing only X at each of d 0 to 21, 0 to 35 and 0 to 42, FCR (*P* < 0.05).

**Table 7:** Main effects of dietary crude protein level (2 levels) and feed additive combination (4 levels) on BW, feed intake and feed conversion ratio among the SBM-free (ALT) treatments; results of 2-way ANOVA.

Item	Additive combination	Crude protein level^1^	BW, g/bird^2^	FI, g/bird	FCR, g:g^2^
**0 to 10 d of age**		‘high’	297	284^b^	1.12^b^
		‘low’	301	295^a^	1.15^a^
SEM			1.49	1.67	0.005
	ALT+PhyG+P+X		297	290	1.15
	ALT+PhyG+P+XB		299	288	1.13
	ALT+PhyG+P+XB+Bet		297	287	1.13
	ALT+PhyG+P+XB+Prob		302	293	1.14
SEM			2.10	2.36	0.007
*P*-value, “crude protein level”			0.086	<0.001	< 0.001
*P*-value “additive combination”			0.180	0.260	0.388
*P*-value “crude protein x additive combination”			0.096	0.500	0.004
**0 to 21 d of age**		“high”	972^b^	988	1.37
		“low”	1,006^a^	1,018	1.37
SEM			6.98	5.73	0.007
	ALT+PhyG+P+X		971^b^	1001^a^	1.40^a^
	ALT+PhyG+P+XB		988^ab^	1009^ab^	1.38^ab^
	ALT+PhyG+P+XB+Bet		985^ab^	980^b^	1.35^b^
	ALT+PhyG+P+XB+Prob		1,013^a^	1018^a^	1.36^b^
SEM			9.87	8.11	0.010
*P*-value, “crude protein level”			0.001	<0.001	0.741
*P*-value “additive combination”			0.011	0.010	0.001
*P*-value “crude protein x additive combination”			0.647	0.245	0.928
**0 to 35 d of age**		“high”	2,158^b^	2,083	1.60^a^
		“low”	2,236^a^	2,087	1.56^b^
SEM			13.99	11.98	0.010
	ALT+PhyG+P+X		2,147^b^	2,081	1.62^a^
	ALT+PhyG+P+XB		2,195^ab^	2,106	1.59^ab^
	ALT+PhyG+P+XB+Bet		2,184^b^	2,049	1.56^b^
	ALT+PhyG+P+XB+Prob		2,262^a^	2,104	1.56^b^
SEM			19.78	16.94	0.014
*P*-value, “crude protein level”			< 0.001	0.803	0.008
*P*-value “additive combination”			0.001	0.068	0.006
*P*-value “crude protein x additive combination”			0.540	0.394	0.858
**30 to 42 d of age**		“high”	2,896^b^	1,354	1.67^a^
		“low”	2,988^a^	1,359	1.64^b^
SEM			15.56	9.88	0.011
	ALT+PhyG+P+X		2,891	1.359	1.69^a^
	ALT+PhyG+P+XB		2,911	1,327	1.67^ab^
	ALT+PhyG+P+XB+Bet		2,943	1,365	1.63^b^
	ALT+PhyG+P+XB+Prob		3,023	1,377	1.63^b^
SEM			22.00	13.97	0.011
*P*-value, “crude protein level”			< 0.001	0.727	0.003
*P*-value “additive combination”			< 0.001	0.083	0.001
*P*-value “crude protein x additive combination”			0.722	0.354	0.748

1 -2.0 to -1.5% points CP vs SBM (control) diet.

2 Measured on the last day of each phase. ^2^FCR values are cumulative, i.e. d 0 to 10, d 0 to 21, d 0 to 35, d 0 to 42. Bet, betaine; BWG, BW gain; FI, feed intake; FCR, feed conversion ratio; PhyG, novel consensus bacterial 6-phytase variant; P, protease; Prob, probiotic; X, xylanase; XB, xylanase–β-glucanase.

### Feed Costs and Carbon Footprint

A comparison of the total feed cost of the diets per kilogram of liveweight gain is presented in Table 6. These calculations were based on market prices of feed ingredients in June 2021 and were inclusive of feed additive costs. Costs were numerically lowest for treatment SBM (0.584 euros/kg BWG) and did not differ significantly from this in treatment ALT+PhyG+P+XB+Prob “low” CP. In all other treatments, feed costs/kg BWG were higher than in the SBM control (*P* < 0.05).

An estimate of the carbon footprint (**CFP**) of the diets per kilogram of liveweight gain, calculated using Wageningen FeedPrint NL software (FeedPrint NL, 2020), is also given in Table 6. All ALT diets had a lower estimated CFP than the SBM control diet (*P* < 0.05) but the total CFP of ALT+PhyG+P+XB+Prob “low” CP was the lowest (1,043 vs. 2,136 g CO_2_ equivalents/kg BWG in the SBM control; *P* < 0.05).

## DISCUSSION

Few previous studies have investigated total SBM replacement in combination with the use of feed additives in poultry. The rationale behind the applied approach was to utilize the unique properties of each included additive for increasing the availability and hence the digestibility of nutrients in the diet (protease to increase AA digestibility, exogenous enzymes xylanase and β-glucanase to enhance the digestibility of NSPs and reduce fiber ANF, betaine to support intestinal functioning and potentially also fiber and protein digestibility and probiotics to support gut health and assist with fiber hydrolysis). The intention was to enable a balanced supply of nutrients to be accessed by birds from the SBM-free ALT diets. Hence, the ALT diets were formulated to provide a similar and well-balanced supply of key nutrients in comparison to the SBM control, except for CP which was reduced in half of the ALT treatments.

The analyzed composition of the alternatives to SBM that were used in this study showed wide variation in the nutrient content of different protein source raw materials. In particular, levels of P varied from 0.12% (in potato protein) to 1.08% (in CSM) and CP varied from 22.4% (in pea flour) to 75.2% (in potato protein). Acid detergent fiber (**ADF**) was markedly higher (by 97 to 738%) in all the alternatives except for pea protein which had a similar ADF content to SBM. This variability is as expected and was beneficial to the design of the trial in being able to combine multiple alternatives together in different proportions in the ALT diet in order to create a diet that was nutritionally balanced and similar to the SBM control (apart from the reduction in CP applied to the “low” CP diet). However, in production practice, the ability to accurately reproduce a nutritional profile comparable to that of a diet based on SBM would require consistency in the nutritional content of each plant alternative over time and yet all ingredients (including SBM) may vary in composition from batch to batch. This variability may be reduced by analyzing each batch of the ingredients being considered for inclusion. In the present study, ingredients were analyzed before feed formulation to ensure that the formulated nutrional level met the requirement and was comparable to that of the respective SBM diet.

Analyzed CP levels in the final diets varied moderately from formulated levels (by a maximum of 10% in the grower phase ALT “low” CP diet). Nevertheless, CP was consistently lower in “low” CP than in “high” CP diet and SBM control diets (by 1.4–3.3% points, across phases). In addition to the oilseed meals (RSM, CSM, SFM) that were included as replacements for SBM, rapeseed oil was used as an alternative source of energy in place of more conventional soybean oil, whilst corn gluten meal, potato protein, pea protein and pea flour were included to increase the CP content of the diets. The bird strain used in the study was a fast-growing commercial broiler strain with high requirements for nutrients. As such, it was expected to provide a sensitive model for evaluating the effects of dietary composition on growth performance outcomes.

The data indicated that birds fed the SBM control outperformed breeder's objectives; final (d 42) body weight and FCR during 0 to 42 d of age were, respectively, 3.8% (120 g) and 1.9% (3 FCR points) above the relevant objectives (Aviagen Inc., 2019). It is likely that the hydrolytic activities of the phytase and xylanase in the SBM control, at the applied dose levels, increased the release of nutrients from phytate and fiber in what was already a highly digestible diet comprising mainly of wheat, corn and SBM with low inclusion of industrial by-products. In contrast, final (d 42) BW was reduced in all ALT treatments whilst overall FCR was maintained by 2 ALT “low” CP treatments, the one containing XB plus Bet and the one containing XB plus probiotics (on top of a background of protease and phytase). The d 42 BW of birds fed ALT+PhyG+P+XB+Prob “low” CP remained slightly below the level achieved by the SBM control (-174 g/bird) but was close to the breeder objective for Ross 308 males (98.3%; Aviagen Inc., 2019). The overall FCR of this treatment was also close to the breeder's objective (99.1%; Aviagen Inc., 2019). These results suggest that the particular combination of added synthetic AA, protease, xylanase, β-glucanase and probiotics present in ALT+PhyG+P+XB+Prob “low” CP (with phytase in the background) could potentially compensate for the 100% replacement of SBM by the plant-based alternatives and about 2% point reduction in CP content of the diet cross phases (based on analyzed values), during an entire growth cycle. Depending on the prevailing cost of feed ingredients, which varies over time, the diet employed in treatment ALT+PhyG+XB+P+Prob “low” CP could also be an economically viable alternative to SBM in some regions, because the production costs (feed cost per kg BWG) associated with treatment ALT+PhyG+P+XB+Prob “low” CP were not significantly different from those of the SBM control. Further, in certain producing regions, the type of diet used in ALT+PhyG+XB+P+Prob “low” CP could offer an environmental sustainability advantage; the total estimated CFP of this diet (including that from fossil fuels, land use and land use change) was markedly lower for ALT+PhyG+P+XB+Prob “low” CP compared to the SBM control. This means that in regions where broiler producers are reliant on the import of SBM from countries that employ land use change as part of the soy production process, there could be a substantial total CFP improvement in switching from a conventional SBM diet to an alternative diet of the type used in ALT+PhyG+P+Prob “low” CP. This would be of little relevance to poultry producers in the US, where the majority of SBM is produced locally without land use change, but is relevant to poultry producers in Europe and Asia, where substantial quantities of SBM are imported from South America where the production process involves land use change.

Most previous studies of 100% SBM replacement have been conducted on slow growing breeds that are not representative of today's breeds that have a high growth rate, lacked statistical power, or used animal protein (fish meal) in the SBM-replacement diet (Hulan and Proudfoot, 1981; Leeson et al., 1987; Tamasgen et al., 2021). In a 6-week study of CSM substitution for SBM at 0, 25, 50, 75, and 100%, Ojewola et al. (2006) showed no significant impairment of weight gain or feed-to-gain ratio in any of the SBM-replacement treatments, although feed intake was increased when 50% or more of SBM was replaced by CSM. Birds fed the control diet containing SBM and no CSM also performed relatively poorly in that study so the comparison is perhaps not fully relevant to today's performance objectives for modern broiler breeds (final average BW was 2,746 g which is 172 g lower than the target as-hatched weight specified for modern breeds such as Ross 308; Aviagen Inc., 2019). Meanwhile, 2 other studies that included treatments where 100% of SBM was replaced by SFM (Rama Rao et al., 2006) and where 75 to 100% of SBM was replaced by RSM (Payvastegan et al., 2017), respectively, observed impaired performance over a 42-d growth cycle, even when metabolizable energy, crude protein and limiting essential AA concentrations were formulated to be maintained constant across diets. Of the few studies that have investigated SBM replacement in conjunction with exogenous enzymes, the past literature is also unclear. Mushtaq et al. (2007) observed that the inclusion of RSM at a level of 30% in the diet as a partial replacement for SBM maintained performance during finisher phase, but BWG was reduced during 1 to 21 d of age and supplementation with a glucanase-xylanase cocktail (presence vs. absence) had no pronounced effect on performance outcomes. Meanwhile, Abdallh et al. (2020) reported that CSM could be included at up to 18% of the finisher diet (up to 6% in starter and 12% in grower) to replace SBM in isocaloric, isonitrogenous wheat/sorghum/SBM-based diets, without any reduction in overall growth performance, although FCR was significantly increased during starter (1–10 d age) and starter-grower (1–24 d of age) phases, in which the highest level of CSM inclusion replaced only 27 to 56% of SBM; protein and energy digestibility were improved by the enzyme combination but this failed to ameliorate the negative effects of high CSM inclusion on FCR. In contrast with these studies, results of the present results suggested that 100% of SBM can be replaced during all growth phases in protein-reduced diets when a combination of exogenous feed additives and probiotics are added to the diet, resulting in overall growth performance outcomes that are close to those produced by a conventional diet based on SBM, and close to breeder objectives.

The 2-way ANOVA that excluded the SBM control showed that reducing the CP content of the SBM-free diets in combination with increased inclusion of synthetic essential AA to balance AA requirements improved growth performance (BW and FCR during 0 to 10d) and increased FI. The capacity to maintain or improve performance with diets that have been reduced in CP by 1 to 3% with added free amino acids has already been demonstrated in several previous studies (van Harn et al., 2019; Macelline et al., 2020). The beneficial effect of reduced CP in the ‘low’ CP treatments in the present study could have been associated with a reduction in the proportion of undigested protein passing into the cecum of birds fed this diet (relative to “high” CP diets). Undigested protein is fermented in the cecum by the cecal microbiota and this results in the production of a variety of metabolites (for example amines, phenols, indoles, cresol and ammonia) that can damage epithelial cells, impair gut morphology induce dysbiosis and infection and reduce growth performance (Wu et al., 2014; Gilbert et al., 2018; Elling-Staats et al., 2022). It is suspected that any such effects were reduced in the “low” CP diets leading to a relatively higher level of growth performance, independent from the effects of the additives.

The 2-way ANOVA also showed that across “low” and “high” CP ALT treatments there was a stepwise improvement in performance (BW at 21, 35, and 42 d of age and FCR during 0–21, 0–35, and 0–42 d of age) with each additional additive included in the diet (XB+probiotics vs. XB or X alone), whereas the one-way ANOVA indicated that this effect was most evident in the “low” CP diets. It is hypothesized that in the “low” CP diets, the broader fibrolytic activity of XB than X alone in resulted in a greater release of NSPs and other nutrients that can become complexed with NSPs in the digesta (Choct et al., 2010), ultimately releasing more energy and NSP-complexed nutrients for maintenance and growth. The calculated diet formulations indicated that the content of total arabinoxylans and beta-glucans was generally higher in the ALT vs. the SBM control diets and it was expected that these constituents provided additional substrate for the X and XB added to the respective ALT treatments. Further addition of the probiotic, which itself has enzymatic activity and can improve growth performance during starter phase as well as gut health and immune responses when co-administered with enzymes (Wealleans et al., 2017; Gibbs et al. 2021), appeared to deliver some further benefit to BW and FCR during early growth phases, but for the overall period its effect was limited. The superior growth performance of birds fed the “low” vs. “high” CP ALT diets does not provide evidence as to the effect of the protease since protease was added to both of these at the same level (800 U/kg). It may be speculated that either the ratio of added protease to indigestible protein was higher in birds fed the “low” CP than in those fed the “high” CP diets, or that the added protease was not sufficient in the “high” CP diets to bring about a material beneficial effect effect on growth performance. A diet mixing error that occurred during diet formulation led to levels of protease in all final diets being well below those intended (800 U/kg compared the 4,000 U/kg that is more commonly applied to broiler diets where beneficial effects on the digestibility of protein and energy have been observed; Romero et al., 2014; Wealleans et al., 2017; Singh et al., 2019). Had ALT+PhyG+P+XB+Prob ‘low’ CP contained 4,000 U/kg of protease, it is possible that an even greater beneficial effect on performance may have been observed, although this remains to be tested.

In other studies in which (partial or total) SBM replacement has failed to maintain growth performance, this has been attributed to the inferior nutritional content of the replacement plant-ingredients including their AA content relative to SBM. Clearly, in the present study, the addition of the exogenous enzymes, betaine and probiotics to the ALT diets was designed to improve nutrient availability. However, differences between the the SBM control and ALT diets in their actual (analyzed) nutrient contents may have impacted on the actual availability of nutrients and energy in the diet. Whilst calculated ME levels were similar across the SBM control and ALT diets, analyzed ADF levels were somewhat higher than intended (formulated) in the “high” CP diets (by 17–42% across phases) and (less so) in the “low” CP diets (by 8–28% across phases). This was likely due to the higher fiber content of RSM, SFM and CSM than SBM. This could have had a suppressing effect on fiber digestibility and feed intake in the ALT treatments even with the activity of the added enzymes, which would explain the observed reduction in overall FI (compared to control) in all ALT treatments except ALT+PhyG+P+XB+Prob “low” CP. It has previously been shown that increasing soluble fiber in the diet increases digesta viscosity and slows the rate of feed passage which can reduce feed intake and the speed of nutrient absorption and utilization for growth (Jha and Mishra, 2021).

The formulated xylanase activity in the ALT treatments containing XB was 1,200 U/kg. This is slightly below the level of 2,000 U/kg used in other studies that have employed the same enzyme (Romero et al., 2014; Singh et al., 2019). Given that growth performance in the ALT treatments containing XB without added betaine or probiotics was still some way below that of the SBM control, the results may indicate that a higher dose level of xylanase or XB is needed in diets containing a higher fiber content. Finally, the analyzed level of Ca was higher in the ALT diets than the SBM control. This could also have had a negative effect on growth performance due to the well-established relationship between high dietary Ca and reduced weight gain and FCR, and negative impact on phytase efficacy (Amerah et al., 2014).

In conclusion, total replacement of SBM with a combination of RSM, SFM, CSM, potato protein, pea protein, pea flour and corn gluten meal, together with supplementation of synthetic AA, xylanase, β-glucanase, protease and probiotics, in a reduced CP diet, maintained overall FCR and FI to a level similar to a CP-normal commercial control diet containing SBM, with phytase in the background. It also achieved overall growth performance outcomes close to broiler breeder objectives at comparable total production costs and a reduced total CFP where the SBM ingredient is imported from regions employing land use and land use change. The use of multiple alternative plant protein sources together with multiple supplemental feed additives may be an effective approach for replacing SBM in commercial broiler diets provided diets are carefully formulated to ensure a normal and balanced supply of essential nutrients.

## DISCLOSURES

Abiodun Bello, Leon Marchal and Yueming Dersjant-Li are employees of Danisco Animal Nutrition & Health (IFF), a global supplier of enzymes.
